# Ultrasound-assisted maillard reaction: a novel strategy for debittering, flavor enhancement, and antioxidant activity improvement of walnut peptides

**DOI:** 10.1016/j.fochx.2026.103958

**Published:** 2026-05-09

**Authors:** Xuehang Wang, Xinyue Li, Mingkai Sun, Hengwen Liu, Yue Leng, Bin Jiang, Jingqi Zhao, Dayong Ren, Ji Wang

**Affiliations:** a College of Plant Protection, College of Mycology, Jilin Agricultural University, Changchun 130118, China; b College of Food Science and Engineering, Jilin Agricultural University, Changchun 130118, PR China

**Keywords:** Ultrasound, Maillard reaction, Walnut-derived peptides, Debittering, Gas chromatography-mass spectrometry, 3-methylbutanal (PubChem CID: 11552), (E)-2-octenal (PubChem CID: 5283324), (Z)-4-heptenal (PubChem CID: 5362814), 2-decanone (PubChem CID: 12741), 3-(methylthio)-2-butanone (PubChem CID: 103788), 1-heptanol (PubChem CID: 8129), 2-pentylthiophene (PubChem CID: 20995), 2-pentylfuran (PubChem CID: 19602), 2-ethylfuran (PubChem CID: 18554), 3-ethyl-2,5-dimethylpyrazine (PubChem CID: 25916)

## Abstract

Walnut peptides (WP) show excellent antioxidant, anti-inflammatory, antihypertensive, and immunomodulatory activities, making them promising for functional foods and nutritional supplements. However, hydrophobic amino acids formed during enzymatic hydrolysis cause severe bitterness, limiting their application.

This study investigated the debittering effect of ultrasound-assisted Maillard reaction (US-MR) on WP by applying ultrasonic pretreatment at different amplitudes followed by Maillard reaction. Results indicated that US-MR promoted WP-glucose conjugation, increased browning intensity and free amino acid content, and enhanced antioxidant activity. Treatment at 30% amplitude achieved the best performance, with the highest fluorescent compound content, the largest reduction in bitter amino acids, and abundant volatile flavors such as aldehydes, ketones, furans, and pyrazines. Sensory evaluation confirmed improved aroma, umami, and texture with significantly reduced bitterness. US-MR thus provides an effective strategy to mitigate WP bitterness and facilitate the development of high-value walnut peptide products.

## Highlights

• Ultrasound-assisted Maillard reaction (US-MR) reduces walnut peptides' bitterness, promotes glucose conjugation, and boosts browning.
• It enriches volatile flavor compounds (aldehydes, ketones, pyrazines) and improves sensory attributes.
• US-MR enhances walnut peptides' antioxidant activity, offering a promising strategy for high-value product development.

## Graphical abstract

**Figure:**
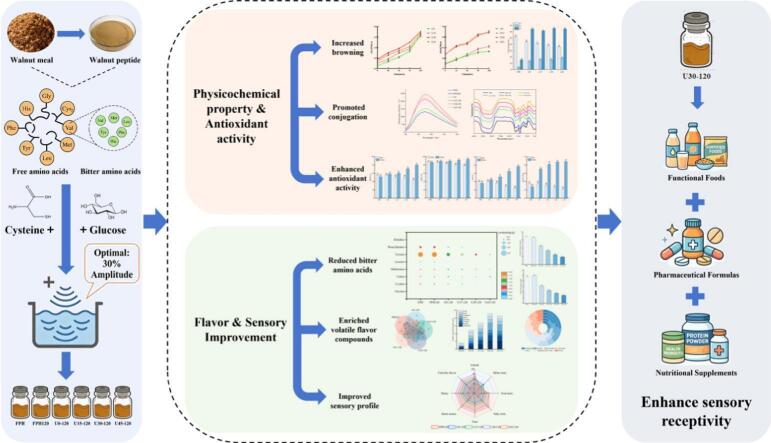

## 1 Introduction

Walnuts, as an important economic crop, are rich in various unsaturated fatty acids, proteins, and vitamins, offering both edible and medicinal value. In recent years, bioactive peptides derived from walnuts have garnered significant attention due to their ease of absorption, multiple health benefits, and lack of toxic side effects (Sheng et al., 2025; Zhang et al., 2025). Walnut peptides (WP) exhibit excellent antioxidant activity and hold great potential in ameliorating cognitive impairments, regulating blood glucose, exerting anti-inflammatory effects, lowering blood pressure, and modulating immune function (Hou et al., 2023; Zheng et al., 2025). These functional properties are closely related to their unique amino acid compositions and sequences. However, the process of protein hydrolysis tends to expose bitter-tasting hydrophobic amino acids, such as phenylalanine (Phe), leucine (Leu), and isoleucine (Ile), etc. (Sun et al., 2022). The side chains of these amino acids are predominantly composed of non-polar groups, which readily bind to the hydrophobic domains of taste receptors, thereby imparting a distinct bitter taste to the hydrolysates (Zhou et al., 2023). This has become a critical technical bottleneck hindering the commercialization of bioactive peptides in the food industry.

The bitterness of peptides arises from the interaction between their molecular structure and human bitter taste receptors (T2Rs). According to current theoretical models, bitter peptides generally comprise two essential functional moieties: a “binding unit” formed by a hydrophobic carbon backbone and a “stimulating unit” containing an α-amino or basic group (Li et al., 2023). When the average distance between these two units is approximately 4.1 Å, they efficiently bind to bitter taste receptors via their hydrophobic recognition domains, thereby activating the bitter taste signaling pathway. The intensity of bitterness depends not only on the nature and spatial arrangement of these functional units but also on the number of hydrophobic residues in the peptide chain; for instance, multiple leucine repeats can significantly enhance bitterness. Furthermore, the bitterness threshold of peptides is influenced by a combination of factors, including amino acid composition, sequence, three-dimensional conformation, and molecular weight (Kim et al., 2008). A higher content of hydrophobic amino acids—such as tryptophan, tyrosine, phenylalanine, and leucine—is typically associated with increased bitterness potency and lower detection thresholds (Sun et al., 2022). The release of bitterness in peptides is governed by both intrinsic protein characteristics and enzymatic hydrolysis conditions. Water-insoluble proteins, being rich in hydrophobic amino acids, are more prone to generate bitter peptides during enzymatic degradation. The cleavage specificity of proteolytic enzymes determines the positioning of hydrophobic residues within the resulting peptides: trypsin tends to place such residues toward the center of the peptide chain, whereas alkaline proteases often expose them at the C- or N- termini that markedly enhance the bitterness of short-chain peptides (Hu et al., 2023). Additionally, the degree of hydrolysis (DH) plays a critical role; low DH primarily yields long-chain bitter peptides, while high DH leads to the release of numerous small, intensely bitter peptides, thereby increasing overall bitterness (Gan et al., 2022). To mitigate bitterness, conventional selective separation techniques—such as organic solvent extraction—can partially remove bitter compounds but are constrained by drawbacks including residual solvents, complex processing steps, and loss of valuable bioactive components, limiting their industrial scalability (Zhu et al., 2019). Consequently, there is growing interest in developing novel debittering technologies that are efficient, safe, and capable of simultaneously improving flavor profiles. Among emerging approaches, ultrasound-assisted Maillard reaction (US-MR) technology has shown particular promise. By enhancing reaction kinetics through physical field effects, this method enables synergistic bitter-masking and flavor enhancement without relying on organic solvents, offering significant potential for practical applications (Yu et al., 2020).

The Maillard reaction (MR) is a non-enzymatic browning reaction between reducing sugars and the free amino groups of amino acids or peptides. It not only serves as the primary pathway for flavor and color formation in food processing but also constitutes an effective strategy for improving the flavor characteristics of protein hydrolysates (Gong et al., 2025). Maillard reaction products (MRPs) are generated through complex chemical pathways. They include volatile substances (e.g., aldehydes, ketones, furans, pyrazines, and sulfur-containing compounds), which contribute pleasant aromas such as baking, meat, and nut flavors. They also contain non-volatile macromolecular substances (e.g., caramelization products) that impart color and help regulate flavor (Nishimura & Abe, 2024). Studies have shown that the MR can effectively enhance the umami, mellow and persistent taste of peptide solutions, which is attributed to the modification of the taste by macromolecular substances and the masking effect of small molecular flavor substances on bitter taste (Ding et al., 2023). Additionally, certain heterocyclic compounds and reduced ketones produced during the reaction possess strong free radical scavenging abilities, which can significantly enhance the antioxidant activity of peptide systems (Ke & Li, 2023).

In recent years, coupling physical field techniques (e.g., ultrasound) with MR has emerged as a new research direction (Yang et al., 2020). High-intensity ultrasonic waves produce cavitation effects in liquid media, triggering local extreme temperature, pressure and strong shear forces, which significantly improve the efficiency of heat and mass transfer within the reaction system and increase the collision frequency between sugars and amino acids/peptides. Meanwhile, the cavitation effect may directly break peptide chains or alter their spatial structures, exposing more reaction sites (e.g., free amino groups), thereby reducing reaction activation energy and accelerating MR from both thermodynamic and kinetic perspectives (Li et al., 2022). Studies have confirmed that the consumption rate of reactants and the formation rate of final products in the ultrasound-assisted glucose-serine model are significantly higher than those in the traditional thermal reactions (Yu et al., 2018). Similarly, spiced beef treated with ultrasound showed a significant increase in both the types and total amounts of key aroma substances, indicating the great potential of ultrasound in promoting flavor formation (Jiang et al., 2024). Therefore, the application of US-MR is expected to efficiently modify and transform the bitterness of peptides, achieve debittering, and provide a reliable technical approach.

US-MR has significant potential for flavor improvement. However, systematic research on how it affects WP's peptide structure, amino acid composition, antioxidant activity, and sensory properties is still lacking. Filling this research gap is critically important for the food industry, as it will provide a comprehensive theoretical framework for applying US-MR to convert bitter, low-value WP hydrolysates into palatable, high-value functional ingredients. Such knowledge is essential for process scale-up and the development of commercial products with improved sensory and bioactive properties. Therefore, this study systematically investigated the effects of US-MR on the Maillard reaction kinetics, peptide structure, flavor profile, antioxidant activity, and sensory attributes of WPs. The objective was to provide an innovative strategy and theoretical support for the debittering and high-value utilization of walnut peptides.

## 2 Materials and methods

### 2.1 Chemicals and materials

Walnut protein isolate (WPI) was provided by the Fermentation Engineering Laboratory of Jilin Agricultural University (Changchun, China). d-glucose, L-cysteine, and glycerol were purchased from Altin Reagent Co., LTD. (Shanghai, China). 1,1-diphenyl-2-picrylhydrazyl (DPPH) and 2, 2′-azino-bis (3-ethylbenzothiazoline-6-sulfonic acid) (ABTS) were purchased from Shanghai Ruiyong Biotechnology Co., LTD. (Shanghai, China). Neutral protease, 1,2-dichlorobene, hydrochloric acid, and phenol were purchased from Beijing Solebo Technology Co., LTD. (Beijing, China). All chemical reagents used in this study were of analytical grade or higher.

### 2.2 Preparation of WP

WPI was extracted from walnut meal powder via the alkaline solubilization-acid precipitation method (Ma et al., 2021), following the procedures below: Walnut meal powder was mixed with deionized water at a solid-to-liquid ratio of 1:40 (*w*/*v*), and the pH of the mixture was adjusted to 12.0. After stirring at 55 °C for 90 min, the mixture was centrifuged at 4500*g* for 15 min. The resulting supernatant was collected and adjusted to pH 4.5 to induce protein precipitation. Following a second centrifugation, the precipitate was freeze-dried to obtain WPI. Subsequently, the WPI was reconstituted into a 2.6% (w/v) solution, homogenized, and denatured in a water bath at 90 °C for 15 min. Neutral protease was added for enzymatic hydrolysis at 50 °C for 3.5 h. These conditions were determined based on our previous optimization studies to achieve a desired degree of hydrolysis. The enzyme was then inactivated by heating at 90 °C for 10 min. The hydrolysate was centrifuged to collect the supernatant, which was adjusted to pH 7.0 and freeze-dried to prepare walnut-derived peptide powder. All samples were stored at −20 °C until further use.

### 2.3 Optimization of key parameters in MR

To systematically investigate the influence of key MR parameters on the WP model, this study used browning intensity as an indicator to monitor the reaction progress. A one-variable-at-a-time approach was employed to examine three factors: heating time, reaction temperature, and sugar-to-peptide mass ratio. Upon completion of the reaction, the samples were rapidly cooled in an ice-water bath to terminate the process. The absorbance was then measured at a wavelength of 420 nm using a UV–Vis spectrophotometer (U3310，Hitachi, Japan) to quantify the degree of browning. On this basis, the response surface method was used to optimize the parameters with the Browning degree as the index.

### 2.4 Preparation of WP by US-MR

WP powder (5%, *w*/*v*), L-cysteine (0.033%, w/v), d-glucose (1.23%, w/v), and glycerol (0.67%, w/v) were dissolved in ultrapure water, mixed thoroughly, and then subjected to ultrasonic treatment in an ultrasonic bath (model KQ-500DE, Kunshan, China; operating frequency 37 kHz; nominal power 500 W) at 25 °C for 30 min with amplitudes of 0% (0 W/L), 15% (30 W/L), 30% (60 W/L), and 45% (90 W/L). These amplitudes were selected based on preliminary experiments to represent varying intensities of ultrasonic cavitation. The bulk solution temperature was maintained at 25 °C to minimize thermal effects and focus on the mechanical and cavitation effects, which are known to generate localized high temperatures and pressures at the micro-scale, thereby promoting reaction kinetics. The system pH was adjusted to 6.4, placed in sealed reaction bottles, and magnetically stirred and heated at 120 °C in an oil bath for 120 min, followed by immediate ice bath cooling to terminate the reaction. The resulting products were labeled as natural flavor models and stored at −80 °C for later use. Samples subjected to ultrasonic treatment were named U0, U15, U30, and U45, respectively; corresponding heated samples were labeled U0–120 to U45–120. Two control groups were set: without added cysteine and glucose, untreated (FPH) and same composition as FPH, heated at 120 °C (FPH120).

### 2.5 Determination of physicochemical indicators MR

To systematically evaluate the MR process, this study employed a multi-indicator joint analysis method. A calibrated pH meter was used to monitor dynamic pH changes (25 ± 1 °C) at reaction times of 0, 30, 60, 90, and 120 min. Browning intensity was determined by measuring the absorbance at 294 nm (intermediate products) and 420 nm (final products) using a UV–Vis spectrophotometer (U3310, Hitachi, Japan). Preliminary experiments verified that 25-fold dilution placed all sample absorbance values within the linear range of the instrument (−2.0 to 4.0 Abs), thus guaranteeing the accuracy and reliability of the measurements. Fluorescence spectra were recorded using a fluorescence spectrophotometer within the excitation wavelength range of 347 nm and emission wavelength range of 360–600 nm (slit width 5 nm), referencing Basso et al.'s method (Basso et al., 2024). Color parameters L (brightness), a* (red/green value), and b* (yellow/blue value) were measured using a calibrated colorimeter (PFX880, Lovibond, China). Simultaneously, structural changes were analyzed using an fourier transform infrared (FTIR) (Nicolet 6700, ThermoFisher, USA) spectrometer (4000–500 cm^−1^, 4 cm^−1^ resolution) with samples prepared via the KBr pellet method (sample: KBr = 1:100).

### 2.6 Determination of free amino acids (FAAs) content

The content of FAAs in the samples was determined using an automatic amino acid analyzer (L-8900, Hitachi, Japan). Briefly, 1.0 g of sample was accurately weighed, followed by the addition of 5 mL of 5% (m/v) sulfosalicylic acid as a protein precipitant. The mixture was vortexed and then diluted to 10 mL with 0.02 M hydrochloric acid. Under nitrogen protection, the sample was subjected to ultrasonication in an ice-water bath for 10 min in pulse mode (5 s sonication, 5 s pause), with the temperature maintained below 4 °C throughout. The resulting suspension was centrifuged at 12,000*g* for 20 min at 4 °C to remove precipitated proteins and large-molecular-weight peptide fragments. The supernatant was collected and adjusted to pH 2.2, followed immediately by centrifugation at 15,000*g* for 15 min at 4 °C to eliminate amino acids prone to isoelectric precipitation (e.g., tyrosine and cysteine). The supernatant was then filtered through a 0.22 μm membrane filter and qualitatively analyzed using the amino acid analyzer. Quantitative analysis of FAAs composition was performed using the external standard method based on peak areas (Gao et al., 2023). Spike recovery experiments revealed that the recovery rates of individual amino acids ranged from 92% to 105%. Control experiments using glycyl-glycine dipeptide showed that the hydrolysis rate of dipeptides was less than 5% under the adopted conditions, confirming that no significant additional FAAs were generated during sample pretreatment.

### 2.7 Analysis of volatile flavor compounds

#### 2.7.1 Extraction of volatile flavor compounds by solid-phase microextraction (SPME)

The extraction of volatile compounds from WP models was studied using SPME. Briefly, 1 μL of 1, 2-dichlorobenzene (internal standard in methanol at 0.1 mg. mL^−1^) was thoroughly mixed into each aliquot of the sample contained in a 5 mL glass vial (20 mL capacity), sealed with a plastic screw cap and a Teflon-coated septum. After pre-equilibration at 50 °C for 20 min in a water bath, aroma compounds were adsorbed for 30 min at 50 °C using a 50/35 μm Carboxen /polydimethylsiloxane/divinylbenzene (CAR/PDMS/DVB) fiber (Supelco Inc., Bellefonte, USA) positioned in the headspace of the vial. The adsorbed fiber was then directly introduced into an Agilent Gas Chromatography (GC) injector for subsequent Gas Chromatography-Mass Spectrometry (GC–MS) analysis (Chiang et al., 2022).

#### 2.7.2 Analysis of volatile flavor compounds by GC–MS

The fiber with absorbed volatile compounds was directly inserted into the 7890B Agilent GC injector port (Agilent Technologies, Santa Clara, California, USA) and desorbed at 250 °C in a splitless mode for 3 min. The separation of volatile compounds was performed using an RT-WAX capillary column (60 × 0.25 mm, 0.25 μm, J & W Scientific, Folsom, CA). Helium was used as the carrier gas at a flow rate of 1.8 mL/min. The initial temperature of the GC column was set at 40 °C and held for 3 min, then increased to 235 °C at a rate of 5 °C/min, and maintained at this temperature for 10 min. The mass spectrometer detector operated with an ion source temperature of 230 °C and an electron voltage of 70 eV. The transfer line temperature was set at 250 °C.Chromatograms were recorded by monitoring the total ion current within the range of 40–450 au (Zhang et al., 2020).

#### 2.7.3 Identification and quantification of volatile flavor compounds

Volatile compounds were identified by comparing their mass spectra with those in the National Institute of Standards and Technology (NIST) mass spectral database. The approximate amount of each volatile compound in the samples was determined by correlating its peak area with that of the internal standard (1, 2-dichlorobenzene) (Chen et al., 2024). The quantification formula is as follows:

(1) $$W_{i}=\frac{A_{i}\times m_{s}}{A_{s}\times m}$$

where *Ai* is the peak area of the compound, *As* is the peak area of the internal standard, *m*_*s*_ is the mass of the internal standard, *m* is the mass of the sample, *Wi* is the concentration of the compound (mg/g)and relative correction factor (assumed value is 1).

### 2.8 Sensory evaluation

The sensory properties of different WP samples were evaluated. The assessment was conducted by a panel consisting of 15 males and 15 females (aged 20–32 years) from the laboratory (Yi et al., 2025). Prior to the evaluation, all panel members were trained to interpret descriptive terms and select appropriate reference solutions for the analysis. Nine sensory attributes (such as roasty, fishy, meaty, umami, mouthfeel, sourness, saltiness, flavor intensity, and bitterness) were selected to evaluate the taste of the peptides. The evaluation was performed using a 1–10 interval scale (0 = none, 10 = extremely strong), with 5 points as the score for the reference solution. Sensory evaluations were conducted in a sensory laboratory equipped to international standards. Then, 5 mL samples (10% *w*/*v* in ultrapure water) were tasted by each panelist in individual sensory booths at room temperature (25 ± 2 °C).

### 2.9 Evaluation of antioxidant activity

Four in vitro chemical analysis methods were employed to systematically assess the antioxidant capacity of the samples. The DPPH method was used to determine free radical scavenging activity: 2 mL of the sample was reacted with an equal volume of 60 μmol/L DPPH solution for 30 min, and absorbance was measured at 517 nm. The Fenton reaction system was used to assess hydroxyl radical scavenging capacity: detection was performed at 510 nm using the Fe^2+^-H₂O₂-salicylic acid reaction system. The ABTS method was used to analyze free radical scavenging performance: 10 μL of the sample was reacted with 190 μL of ABTS^+^ working solution for 6 min, and absorbance was measured at 734 nm. The iron cyanide reduction method was used to assess reducing power: the reaction system was water-bathed at 50 °C for 30 min, centrifuged after adding trichloroacetic acid, and the supernatant was reacted with FeCl₂, with absorbance measured at 700 nm. All experiments used Vc as a positive control, and the free radical scavenging rate was calculated using the following formula:

(2) $$\text{clear rate}\%=\frac{A_{0}-\left(A_{1}-A_{2}\right)}{A_{0}}\times 100\%$$

where: Blank sample absorbance value with distilled water instead of sample solution; Absorbance value with sample solution added; Absorbance value of sample background; Absorbance value of solution without salicylic acid.

### 2.10 Determination of total sulfhydryl (SH) content

The total content of thiol groups (SH) in the untreated and ultrasonic-treated sample solutions was determined by the modified Ellman (DTNB) method (Žilić et al., 2012). In brief, 1 mg/mL of the sample solution was diluted 25 times with a buffer solution. The sample was incubated at 55 °C in Tris-HCl buffer (pH 8.0) containing dithiothreitol (DTT) for 40 min until complete denaturation and reduction. The insoluble impurities were removed by centrifugation, and 1 mL of the supernatant was taken. 0.25 mL of 10 mmol/L DTNB solution was added, and the reaction was carried out at room temperature in the dark for 30 min. The absorbance was measured at 412 nm using an UV–Vis spectrophotometer (U3310, Hitachi, Japan), and the thiol concentration was calculated using the molar absorptivity of 1.36 × 10^4^ M^−1^ cm^−1^.

### 2.11 Statistical analysis

All data were expressed as mean ± standard deviation (*n* = 3) and analyzed by one-way analysis of variance (ANOVA) followed by Tukey's test using SPSS 23.0 software. Tukey's test was employed to determine *p*-values. All figures were generated using Origin 2021 software, and a p-value <0.05 was considered statistically significant.

## 3 Results and discussion

### 3.1 Effect of key reaction parameters on browning intensity

**Fig. 1:**
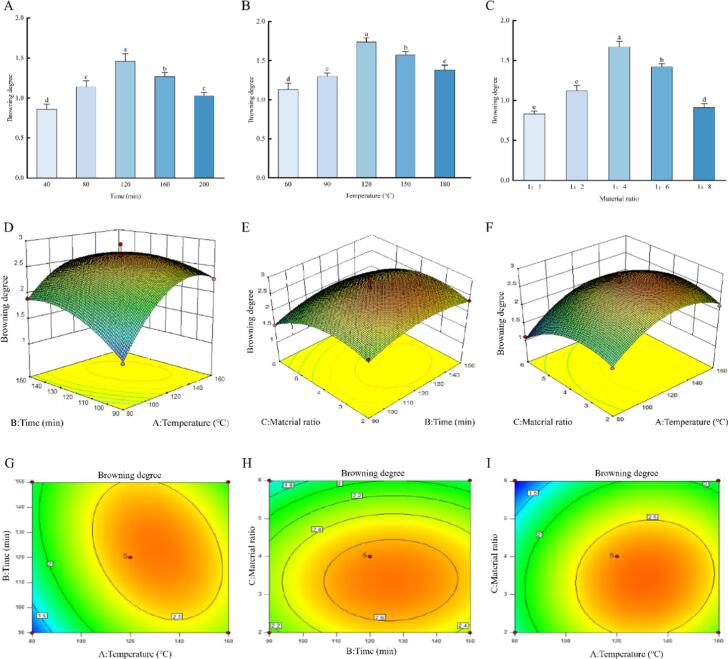
Key parameters influencing the browning intensity of the Maillard reaction (MR) system. (A) Effect of heating time on browning intensity; (B) effect of temperature on browning intensity; (C) effect of sugar-to-peptide ratio on browning intensity; (D, G) 3D surface and contour plots of time and temperature; (E, H) 3D surface and contour plots of time and material ratio; (F, I) 3D surface and contour plots of temperature and material ratio. Different letters indicate significant differences between group means (*p* < 0.05).

This study systematically investigated the combined effects of heating time, temperature, and sugar-peptide ratio on the browning intensity of the MR system through single-factor and response surface methodology (RSM) experiments. The results of the single-factor analysis indicated that the browning degree showed a trend of increasing first and then decreasing with the extension of heating time, reaching a peak at 120 min (Fig. 1A), mainly due to the continuous generation of brown polymers during the reaction's middle and later stages. Further extension of the heating time might lead to a decrease in browning due to the consumption of reactants or degradation of products. The influence of temperature on the reaction kinetics was also significant: below 120 °C, heating could promote the initial condensation and rearrangement steps, thereby enhancing browning; while above 120 °C, browning decreased (Fig. 1B), possibly related to the formation of volatile substances or the decomposition of melanoidins (Zhang et al., 2024). The sugar-peptide ratio also played a crucial role in regulating the reaction pathway, with a ratio of 1:4 being most favorable for the formation of color-forming intermediates. Excessive sugar may cause changes in reaction equilibrium or polymerization patterns, thereby reducing browning efficiency (Fig. 1C). RSM analysis further revealed the interactive effects among the three factors (Fig. 1D-I). The interaction between time and temperature was the most significant: when one factor was fixed, the browning degree showed a single-peak trend (first increasing and then decreasing) with the increase of the other factor, reaching the maximum at 120 min and 120 °C. In contrast, the influence of the sugar-peptide ratio was relatively weak: when the sugar-peptide ratio was fixed, the browning degree still exhibited a peak change with temperature or time; while when temperature or time was fixed, the browning degree only increased gradually with the increase of the sugar-peptide ratio. This indicates that within the experimental range, time and temperature are the key factors dominating the browning process, and their effects are significantly greater than that of the sugar-peptide ratio.

The optimal process conditions predicted by the model were: heating time of 120 min, temperature of 120 °C, and sugar-peptide ratio of 1:4. The browning degree obtained from verification experiments under these conditions was highly consistent with the predicted value, confirming the reliability of the model. In summary, the effects of time and temperature on browning intensity exhibit a non-linear dual role of promotion and inhibition, which is consistent with the accumulation mechanism of melanoidins in MR and the trend of side reactions occurring under high temperature or prolonged heating conditions. This study clarified the interactive relationships among key parameters of MR in the WP system, providing a theoretical basis and process guidance for the controlled regulation of MR in WP-based products.

### 3.2 Effect of US-MR on pH value

**Fig. 2:**
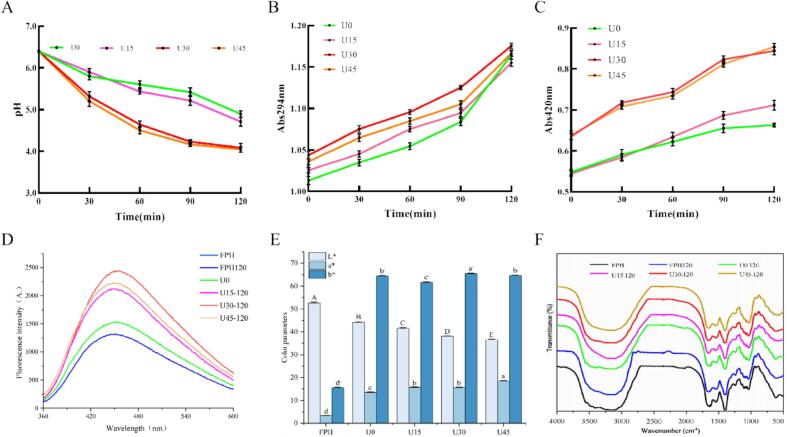
Effect of US-MR on the chemical composition of WP. (A) Changes in pH of WP during heating; (B) changes in absorbance (294 nm) of WP during heating; (C) changes in absorbance (420 nm) of WP during heating; (D) changes in fluorescence intensity of WP during heating; (E) changes in color of WP during heating; (F) FTIR spectroscopy of WP. Different letters indicate significant differences between group means (*p* < 0.05).

The pH value is one of the key parameters for monitoring the progression of the MR. Fig. 2A shows the changes in pH values of ultrasound-pretreated WP models (U15, U30, and U45) and the control group (U0). At *t* = 0, there was no significant difference in pH values between the control and ultrasound-pretreated WP models, indicating that ultrasound pretreatment itself had no significant impact on the initial pH of the system. With the extension of heating time, the pH reduction in ultrasound-treated samples was significantly greater than that in the control group (U0) (*p* < 0.05), and the degree of pH decrease was positively correlated with ultrasound intensity. Among all samples, U45 exhibited the most significant pH reduction at the end of the reaction, followed by U30, U15, and U0 in sequence. This phenomenon suggests that the cavitation effect induced by ultrasound promotes the isomerization of glucose, thereby enhancing the reactivity of reducing sugars. Additionally, ultrasound pretreatment can effectively homogenize the WP model system, achieving efficient heat and mass transfer and increasing the formation rates of MR intermediates and end products (Yan et al., 2024). Meanwhile, the decrease in pH may be attributed to two main factors: the generation of organic acids (e.g., acetic acid, formic acid, hexanoic acid, heptanoic acid, and octanoic acid) during MR, and the reduction in free N-terminal amino groups of amino acids and peptides (Geng et al., 2019). These changes not only reflect the extent of MR progression but are also closely associated with the formation of subsequent flavor substances. This finding is consistent with the results of Yu et al., who observed a similar pH decrease in a soybean meal hydrolysate/xylose/cysteine model during heating, further verifying the applicability of ultrasound treatment in accelerating MR (Yu et al., 2018).

### 3.3 Effect of ultrasonic pretreatment on browning intensity

Browning intensity variation serves as a critical indicator for tracking the progression of the MR. In this study, the formation dynamics of MR intermediates and final products in WP models during thermal treatment were monitored by determining the absorbance values at 294 nm and 420 nm. As illustrated in Fig. 2B and C, at the initial time point (*t* = 0), the ultrasound-pretreated samples (U15, U30, and U45) exhibited higher absorbance at 294 nm compared with the control group (U0). This observation suggests that ultrasound pretreatment facilitated the generation of UV-absorbing MR intermediates at the early stage of the reaction. In contrast, no significant differences in absorbance at 420 nm were detected among all samples at t = 0, verifying that ultrasound treatment alone did not trigger the formation of MR final products such as melanoidins. With the prolongation of heating time, the absorbance at both wavelengths increased continuously across all samples, accompanied by a marked accumulation of MR intermediates and browning pigments (melanoidins). This trend confirms the progressive intensification of the MR during thermal processing. Notably, the ultrasound-treated groups especially U30 and U45 showed significantly higher absorbance at 294 nm and 420 nm than the control group. These results demonstrate that ultrasound pretreatment effectively accelerates the entire MR process, leading to elevated yields of both intermediate species and final melanoidin products. The underlying mechanism may be attributed to the conformational modifications of WP induced by ultrasound: specifically, ultrasound treatment reduces the α-helix content, increases the proportion of random coil structures, enhances molecular flexibility, and elevates the frequency of intermolecular collisions, thereby improving the efficiency of glycosylation reactions (Sun et al., 2025). Consistent with our findings, Ye et al. also reported that high-intensity ultrasound can expedite the formation of MR intermediates and melanoidin products at relatively low processing temperatures (55 °C and 60 °C), which provides a promising technical basis for the advancement of low-temperature food processing technologies (Ye et al., 2022).

### 3.4 Effect of ultrasonic pretreatment on fluorescence intensity

Changes in fluorescence intensity provide crucial insights into the chemical evolution during the intermediate stage of MR. This study observed that MR can generate fluorescent compounds before the formation of brown polymers. The fluorescence spectra of the WP models are shown in Fig. 2D. The emitted fluorescence of the samples ranged between 360 and 600 nm, a characteristic consistent with the complex fluorophore structures formed during MR. The control groups (FPH and FPH120) without added sugar and cysteine did not exhibit significant changes in fluorescence intensity before and after heating, indicating that simple thermal treatment cannot induce the formation of fluorescent compounds. The increase in ultrasound amplitude of the WP in the groups with added sugar and cysteine (U0–120, U15–120, U30–120, and U45–120) significantly enhanced the fluorescence intensity of the MR compared with FPH120. This confirms that the generation of fluorescent compounds is indeed a characteristic phenomenon of MR. Notably, sample U30–120 exhibited the highest fluorescence intensity at 450 nm, indicating the formation of a large amount of fluorescent compounds. Notably, sample U30–120 exhibited the highest fluorescence intensity at 450 nm, indicating the formation of a large amount of fluorescent compounds. This phenomenon may be due to the optimal cavitation intensity produced by 30% amplitude sonication, which effectively promotes the interaction between the reactive molecules and prevents compound degradation (Sun et al., 2025). The relatively lower fluorescence intensity in sample U45–120 can be attributed to the excessive ultrasound treatment affecting the stability or formation pathway of fluorescent compounds. Overly intense cavitation may generate reactive species (e.g., hydroxyl radicals) that could degrade nascent fluorescent intermediates or promote their rapid conversion into non-fluorescent advanced Maillard reaction products, such as melanoidins. This phenomenon is consistent with previous reports where excessive ultrasonic energy led to the degradation of reaction products (Sun et al., 2025). This result is consistent with the observed enhancement of fluorescence intensity in MRPs extracted from soy protein hydrolysates (Huang et al., 2022). Additionally, it has been reported that fluorescent compounds derived from MR can generate hydrogen atom radicals. These results suggest that the fluorophore compounds generated during MR of protein hydrolysate-glucose may enhance the antioxidant activity of WPs (Gómez-Estaca et al., 2021).

### 3.5 Effect of ultrasonic pretreatment on color

MR and caramelization are two primary non-enzymatic reactions that cause color changes in food during heating. As shown in Fig. 2E and Table 1, significant changes in color parameters were observed in ultrasonically pretreated samples (U15, U30, and U45) compared to the control sample (U0) (*p* < 0.05). At *t* = 0, the a* and b* parameter values gradually increased with increasing ultrasound intensity, while the L* value decreased, confirming the occurrence of MR during ultrasonic pretreatment. Simultaneously, it was found that the a* and b* parameter values of all WP models significantly increased (*p* < 0.05) with prolonged heating time, with more pronounced increases observed in U30 and U45 samples. The increase in a* and b* parameter values may be related to the formation of various brown compounds through MR (Liu et al., 2010). However, when the heating time was extended from 90 to 120 min, a slight decrease in the b* value was observed in U30 and U45 samples, indicating the degradation of colored compounds. On the other hand, the L* value decreased with increasing heating time, indicating the formation of darker-colored compounds. Interestingly, the U15, U30, and U45 samples exhibited deeper colors compared to the U0 sample (control). These findings are consistent with the changes in browning intensity (Fig. 2B and C), collectively confirming that ultrasound treatment has a reinforcing effect on MR. Similarly, Corzo-Martínez et al. also reported an increase in advanced MR products in models subjected to ultrasonic treatment at 50% amplitude and 40 °C (Corzo-Martínez et al., 2014).

**Table 1:** Effect of US-MR on color change of WP.

Heating time(min)						
Parameters	Samples	0	30	60	90	120
L*	FPH	52.57 ± 0.28^c^	51.86 ± 0.20^d^	51.08 ± 0.16^ef^	52.86 ± 0.29^bc^	52.66 ± 0.32^c^
	U0	51.59 ± 0.13^de^	53.41 ± 0.16^ab^	45.45 ± 0.23^h^	43.56 ± 0.19^ij^	44.15 ± 0.32^i^
	U15	51.56 ± 0.29^de^	53.56 ± 0.19^a^	43.26 ± 0.20^j^	39.30 ± 0.13^n^	41.52 ± 0.26^m^
	U30	50.51 ± 0.25^f^	49.41 ± 0.45^g^	42.51 ± 0.25^kl^	41.97 ± 0.09^lm^	38.16 ± 0.03^o^
	U45	50.49 ± 0.26^f^	49.34 ± 0.30^g^	42.52 ± 0.22^kl^	42.59 ± 0.28^k^	36.60 ± 0.24^p^
a*	FPH	3.80 ± 0.10^m^	3.26 ± 0.10^n^	3.45 ± 0.19^mn^	3.44 ± 0.22^mn^	3.34 ± 0.12^mn^
	U0	3.37 ± 0.20^mn^	4.32 ± 0.10^l^	11.52 ± 0.16^f^	13.52 ± 0.25^d^	13.48 ± 0.20^d^
	U15	3.58 ± 0.16^mn^	5.27 ± 0.10^k^	12.63 ± 0.13^e^	14.53 ± 0.30^c^	15.70 ± 0.30^b^
	U30	6.73 ± 0.20^i^	7.49 ± 0.22^h^	12.48 ± 0.22^e^	14.48 ± 0.21^c^	15.60 ± 0.20^b^
	U45	6.21 ± 0.09^j^	8.25 ± 0.14^g^	11.60 ± 0.16^f^	13.52 ± 0.16^d^	18.49 ± 0.22^a^
b*	FPH	13.95 ± 0.31^k^	14.60 ± 0.26^k^	14.41 ± 0.27^k^	15.45 ± 0.17^j^	15.60 ± 0.19^j^
	U0	15.78 ± 0.34^j^	51.37 ± 0.40^h^	62.18 ± 0.14^de^	62.67 ± 0.30^d^	64.43 ± 0.24^c^
	U15	17.70 ± 0.32^i^	52.34 ± 0.26^g^	62.56 ± 0.26^d^	62.70 ± 0.32^d^	61.57 ± 0.30^e^
	U30	17.37 ± 0.16^i^	54.41 ± 0.39^f^	65.41 ± 0.16^b^	66.37 ± 0.26^a^	65.46 ± 0.18^b^
	U45	17.13 ± 0.16^i^	52.28 ± 0.21^g^	65.56 ± 0.24^b^	66.74 ± 0.28^a^	64.53 ± 0.26^c^

Values are given as mean ± SD (n = 3). Values followed by different superscript letters a, b, c are significantly different (*p* < 0.05).

### 3.6 Effect of ultrasonic pretreatment and heating on FTIR spectra

FTIR spectroscopy was used to investigate the effects of ultrasonic pretreatment and heating on the interactions between glucose and polypeptides. Fig. 2F shows the FTIR spectra of the heated samples. Compared to its unheated counterpart (FPH), the FTIR spectrum of the FPH120 sample exhibited slight differences, particularly in the amide A and B regions, indicating a significant impact of the heating process on the peptide structure. After the heating process, the spectra of the WP models (U0–120, U15–120, U30–120, and U45–120) showed significant differences from those of the FPH and FPH120 samples. A significant decrease in transmittance intensity was observed in the amide A and B regions (3380–3340 cm^−1^), indicating cross-linking of free -OH groups in the peptides after heating. Additionally, a more pronounced decrease in transmittance intensity was observed in ultrasonically treated WP (U15–120, U30–120, and U45–120), suggesting that ultrasonic pretreatment promoted the binding of free -OH peptide groups to glucose. Similarly, a significant decrease in transmittance intensity was observed in the C—O stretching (1600–1700 cm^−1^) of amide I after heating. A similar trend was observed in the N—H bending deformation (1500–1550 cm^−1^) of amide II. Furthermore, noticeable deformations in the C—N stretching and N—H bending vibrations of amide III (1220–1400 cm^−1^) were noted after heat treatment, particularly in the ultrasonically pretreated WP models. The lowest transmittance intensity for amide III vibrations was observed in the U30–120 sample, followed by U45–120, U15–120, and U0–120. The more significant reduction in transmittance intensity for C=O, C—N, N—H, and -OH stretching vibrations in ultrasonically treated WP samples points to a higher degree of polypeptide cross-linking in these groups. The accelerated MR induced by ultrasonic treatment may be attributed to the intense shear forces generated during ultrasonication, which enhance the homogeneity of the WP system, promote efficient heat and mass transfer, and thereby elevate the cross-linking rate during thermal processing. Similar observations have been reported by Sun et al., who demonstrated that ultrasonic treatment significantly accelerates the cross-linking between glutathione and xylose during heat treatment (Sun et al., 2019). Collectively, the FTIR analysis confirms the formation of covalent cross-links between WP polypeptides and sugars during thermal processing, and further verifies that ultrasonic pretreatment serves as an effective strategy to accelerate this reaction process.

### 3.7 Effect of ultrasonic pretreatment on FAAs content

**Fig. 3:**
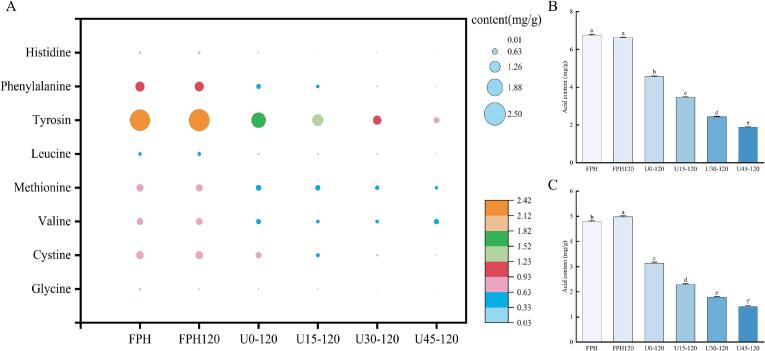
Effects of US-MR on amino acid content of WP. (A) Bubble plot of free amino acid content in WP; (B) total free amino acid content in WP; (C) total bitter amino acid content. Different letters indicate significant differences between group means (*p* < 0.05).

FAAs act as pivotal non-volatile flavor compounds, whose composition and concentration directly modulate the sensory attributes of peptide-based products. The FAAs content of the WP samples is presented in Table 2 and Fig. 3. The decline in FAAs content during thermal processing is primarily ascribed to two key pathways: first, the cross-linking reaction between glucose molecules and amino acid residues of FAAs; second, the Strecker degradation and thermal decomposition of amino acids that occur concomitantly during the reaction (Wang et al., 2025). Notably, ultrasonic pretreatment further accelerated the depletion of FAAs, which provides direct evidence that ultrasonication promotes the MR between FAAs residues and sugar molecules. These results are consistent with the browning intensity data (Fig. 2B and C), where a lower FAAs content correlates positively with a higher browning intensity. This correlation can be explained by the fact that the active free radicals generated by ultrasound can oxidize the amino groups of amino acids—especially those of lysine and glycine—to form highly reactive aldehyde intermediates. Meanwhile, ultrasound enhances the frequency of intermolecular collisions, thereby facilitating the condensation reaction between sugar and amino acid molecules (Kang et al., 2016). In contrast to the glucose-added WP samples, the total FAAs content in the sugar-free FPH120 sample exhibited an increase after heating. This phenomenon may be attributed to the cleavage of peptide chains induced by thermal treatment, which generates new free amino acid molecules. From a flavor perspective, the variation trends of umami and bitter FAAs were consistent with that of total FAAs. Specifically, the content of bitter FAAs decreased significantly across all WP samples. Among these, the U30–120 group showed the most pronounced reduction, followed in sequence by U15–120, U45–120, and U0–120. While U45–120 exhibited a higher amplitude than U30–120, its slightly less pronounced reduction in bitter FAAs may be attributed to an “over-processing” effect. Excessive ultrasonic cavitation may generate reactive radicals that could promote the re-generation of some amino acids from degraded peptide fragments or shift the reaction pathway, leading to a less efficient net consumption of bitter FAAs. Given that the bitterness of the final product is closely related to the content of bitter FAAs, the ultrasound-mediated reduction in bitter FAAs levels is of great significance for improving the food acceptability of WP-derived products.

**Table 2:** Effect of US-MR on free amino acid content of WPs (mg/g sample).

Amino acids	FPH	FPH120	U0–120	U15–120	U30–120	U45–120
Glycine	0.227 ± 0.033^a^	0.192 ± 0.043^ab^	0.130 ± 0.032^bc^	0.113 ± 0.019b^cd^	0.062 ± 0.015^cd^	0.034 ± 0.009^d^
Cystine	0.845 ± 0.019^a^	0.835 ± 0.026^a^	0.660 ± 0.032^b^	0.434 ± 0.026^c^	0.222 ± 0.030^d^	0.109 ± 0.016^e^
Valine	0.718 ± 0.039^a^	0.722 ± 0.026^a^	0.555 ± 0.020^b^	0.416 ± 0.015^c^	0.414 ± 0.020^c^	0.576 ± 0.026^b^
Methionine	0.778 ± 0.032^a^	0.762 ± 0.032^a^	0.625 ± 0.024^b^	0.581 ± 0.032^b^	0.459 ± 0.016^c^	0.364 ± 0.017^d^
Leucine	0.398 ± 0.015^a^	0.441 ± 0.029^a^	0.206 ± 0.015^b^	0.150 ± 0.023^bc^	0.147 ± 0.025^c^	0.096 ± 0.012^d^
Tyrosin	2.341 ± 0.042^a^	2.412 ± 0.028^a^	1.671 ± 0.026^b^	1.253 ± 0.034^c^	0.977 ± 0.034^d^	0.633 ± 0.023^e^
Phenylalanine	1.069 ± 0.024^a^	1.055 ± 0.019^a^	0.541 ± 0.053^b^	0.370 ± 0.035^c^	0.155 ± 0.041^d^	0.070 ± 0.012^d^
Histidine	0.258 ± 0.032^a^	0.261 ± 0.017^a^	0.163 ± 0.015^b^	0.105 ± 0.009^c^	0.091 ± 0.017^c^	0.031 ± 0.005^d^
Total aa	6.761 ± 0.038^a^	6.624 ± 0.023^a^	4.560 ± 0.038^b^	3.454 ± 0.053^c^	2.435 ± 0.032^d^	1.884 ± 0.035^e^

For each amino acid, values followed by different lowercase superscript letters indicate statistically significant differences (*p* < 0.05).

### 3.8 Effect of ultrasonic pretreatment on volatile flavor compounds

**Fig. 4:**
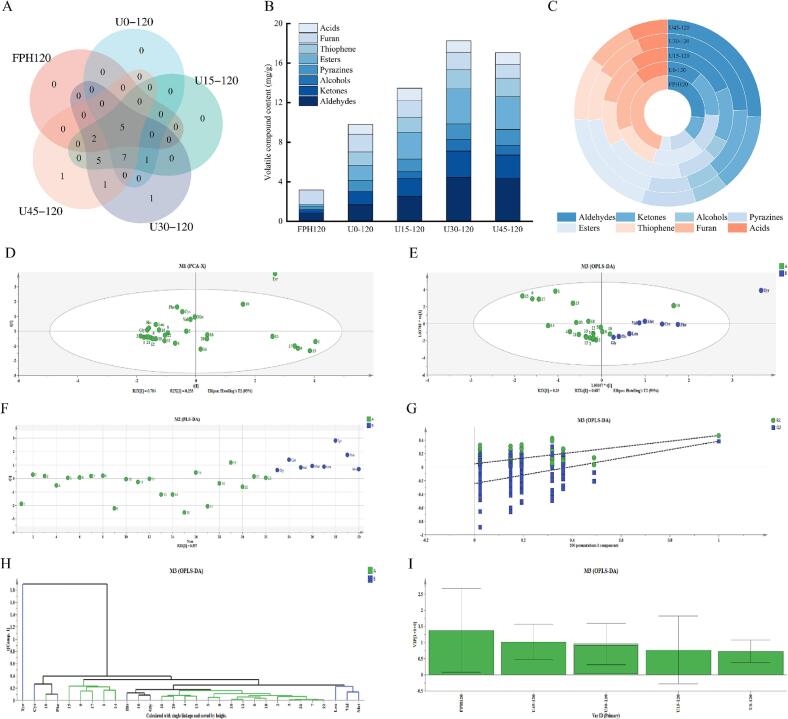
Comparison of volatile compounds in WP. (A) Venn diagram of 22 volatile compounds; (B) total content of different classes of volatile compounds; (C) relative content of different classes of volatile compounds; (D) principal component analysis (PCA) score plot; (E, F) orthogonal partial least squares-discriminant analysis (OPLS-DA) and partial least squares-discriminant analysis (PLS-DA) score plots based on amino acid composition; (G) 200-permutation test for the partial least squares regression (PLSR) model; (H) variable importance in projection (VIP) scores of the PLSR model; (I) box plot of VIP scores across different groups.

It is widely believed that ultrasonic pretreatment accelerates the MR rate. However, studies on the impact of ultrasonic treatment on the aroma profile of peptide/sugar model systems are scarce. To investigate the mechanism of ultrasonic pretreatment on aroma compounds, this study conducted a systematic analysis of aroma distribution using different WP models total of 22 volatile compounds, including aldehydes, ketones, alcohols, thiophenes, furans, pyrazines, esters, and acids, which may potentially influence the aroma of peptides, were identified and quantified (Table 3). The Venn diagram revealed the detection of 7, 13, 20, 22, and 21 volatile compounds in FPH120, U0–120, U15–120, U30–120, and U45–120, respectively (Fig. 4A). Additionally, seven common volatile compounds, predominantly aldehydes, were identified in all samples, confirming that the types of volatile compounds in WP showe no significant changes after MR, with aldehydes remaining the primary components of their flavor characteristics (Yang et al., 2023). As shown in Fig. 4B, the presence of reducing sugars in the reaction system significantly increased the total volatile compound content. Specifically, the highest total volatile compound content was observed in U30–120 and U45–120 samples (18.247 mg/g and 17.064 mg/g, respectively). Fig. 4C demonstrated that the relative acid content in U30–120 decreased by 0.68% compared to U45–120. An increase in acid content may lead to undesirable flavors such as sweatiness or rancidity. The relative aldehyde content increased by 1.26%. Similarly, (Yuan et al., 2020) reported that enzymatic MR preparation of aromatic rapeseed oil resulted in higher relative aldehyde content compared to traditional oils. Aldehydes are a key source of fat flavor and play a significant role in WP, indicating that appropriate ultrasonic assistance can enhance their flavor profile.

**Table 3:** Effect of US-MR on volatile flavor compounds in WP (mg/g sample).

Code		RT (min)	FPH120	U0–120	U15–120	U30–120	U45–120
	**Aldehydes**						
1	3-methylbutanal	2.34	0.837 ± 0.038^d^	0.913 ± 0.028^d^	1.110 ± 0.038^c^	2.763 ± 0.066^b^	2.920 ± 0.032^a^
2	Heptaldehyde	9.54	0^b^	0^b^	0^b^	0.213 ± 0.05^a^	0^b^
3	(Z)-4-Heptenal	14.21	0^b^	0^b^	0^b^	0.203 ± 0.020^a^	0.177 ± 0.041^a^
4	(E)-2-Octenal	19.45	0^d^	0^d^	0.433 ± 0.433^c^	0.537 ± 0.537^b^	0.763 ± 0.763^a^
5	Methylal	20.12	0^c^	0.760 ± 0.040^a^	0.667 ± 0.058^a^	0.273 ± 0.035^b^	0.180 ± 0.038^b^
6	2-Undecenal	26.19	0^c^	0^c^	0.180 ± 0.040^b^	0.280 ± 0.040^a^	0.163 ± 0.029^b^
7	Trans-2-Decenal	28.56	0.020 ± 0.006^b^	0^b^	0.117 ± 0.020^a^	0.177 ± 0.030^a^	0.170 ± 0.032^a^
	**Ketones**						
8	3-(Methylthio)-2-Butanone	15.23	0.330 ± 0.032^b^	0^c^	0.353 ± 0.038^b^	0.537 ± 0.032^a^	0.343 ± 0.055^b^
9	2-Decanone	22.12	0^d^	1.327 ± 0.058^c^	1.463 ± 0.047^c^	2.127 ± 0.052^a^	1.980 ± 0.058^b^
	**Alcohols**						
10	1-Heptanol	21.45	0^c^	0^c^	0.237 ± 0.043^b^	0.360 ± 0.040^a^	0.247 ± 0.030^b^
11	1-Octen-3-ol	27.89	0^c^	0^c^	0.337 ± 0.032^b^	0.470 ± 0.055^a^	0.430 ± 0.035^ab^
12	2-Undecen-1-ol	31.23	0^c^	0^c^	0.117 ± 0.020^b^	0.330 ± 0.032^a^	0.320 ± 0.047^a^
	**Pyrazines**						
13	3-Ethyl-2,5-Dimethylpyrazine	20.14	0.347 ± 0.038^d^	1.140 ± 0.042^c^	1.280 ± 0.032^b^	1.580 ± 0.042^a^	1.607 ± 0.049^a^
	**Esters**						
14	Phenethyl Hexanoate	21.34	0^c^	0.020 ± 0.006^c^	0.553 ± 0.035^b^	1.227 ± 0.064^a^	1.137 ± 0.044^a^
15	Octyl Formate	25.12	0^e^	1.230 ± 0.061^d^	1.790 ± 0.046^c^	2.253 ± 0.046^a^	2.077 ± 0.035^b^
16	Butyl Triacontanoate	40.19	0.160 ± 0.032^bc^	0.260 ± 0.040^ab^	0.347 ± 0.062^a^	0.087 ± 0.020^c^	0.090 ± 0.021^c^
	**Thiophene**						
17	2-Pentylthiophene	21.11	0^d^	1.363 ± 0.038^c^	1.540 ± 0.059^b^	1.900 ± 0.049^a^	1.877 ± 0.023^a^
	**Furan**						
18	2-Ethylfuran	2.13	0.243 ± 0.050^c^	0.463 ± 0.047^b^	0.853 ± 0.048^a^	0.753 ± 0.041^a^	0.763 ± 0.027^a^
19	2-Pentylfuran	10.23	1.227 ± 0.052^a^	1.337 ± 0.032^a^	0.823 ± 0.038^c^	1.040 ± 0.042^b^	0.640 ± 0.049^d^
	**Acids**						
20	Acetic acid	20.78	0^b^	0.653 ± 0.048^a^	0.743 ± 0.024^a^	0.757 ± 0.045^a^	0.773 ± 0.061^a^
21	Heptanoic acid	35.29	0^c^	0.060 ± 0.012^bc^	0.087 ± 0.026^b^	0.160 ± 0.029^a^	0.230 ± 0.032^a^
22	Octanoic acid	37.99	0^c^	0.263 ± 0.035^b^	0.440 ± 0.040^a^	0.220 ± 0.058^b^	0.177 ± 0.026^b^

Values are expressed as mean ± SD (n = 3). Within the same row, values followed by different lowercase superscript letters indicate significant differences (*p* < 0.05).

Aldehydes, as the primary aroma components in WP, were significantly affected by ultrasonic treatment in terms of their composition and content. Seven types of aldehydes were detected in the WP samples, with 3-methylbutanal and (E)-2-octenal being the predominant aldehydes in the U30–120 and U45–120 samples, while 3-methylbutanal and methylbutanal were the main aldehydes in the U0–120 and U15–120 samples. Confirming that the primary classes of volatile compounds in WP show no significant changes after MR, with aldehydes remaining the dominant class. However, within this class, ultrasonic treatment significantly increased the diversity and concentration of individual aldehyde compounds. Specifically, various aldehydes not observed in the control group (such as (Z)-4-heptenal and trans-2-decenal) were detected in the ultrasonic group. The increase in aldehydes was attributed to the ultrasonic cavitation effect promoting lipid oxidation to form hydroperoxides, which further degraded into aldehydes during heating (Jiang et al., 2024), while ultrasonic treatment also enhanced the Strecker degradation pathway of amino acids. The increase in aldehyde content was dependent on the ultrasonic energy density: at 60 W/L (corresponding to 30% amplitude), aldehyde content peaked, while at the higher energy density of 90 W/L (45% amplitude), a significant decrease in aldehyde content was observed. This suggests a dose-dependent effect, where excessive ultrasonic energy may cause the transformation or decomposition of aldehydes, highlighting the importance of optimizing ultrasound intensity for flavor development. Although ketones were present at lower concentrations, their content significantly increased with ultrasonic intensity (*p* < 0.05), primarily consisting of trans-2-decanone and 3-(methylthio)-2-butanone. The specific presence of 1-octen-3-ol, 1-heptanol, and 2-undecen-1-ol in alcohols confirmed the promoting effect of ultrasonication on the degradation of unsaturated fatty acids. Among them, 1-octen-3-ol imparted a mushroom-like aroma to the system, and as ultrasonic amplitude increased, the alcohol content in the flavor model significantly rose (*p* < 0.05).

In terms of sulfur-containing heterocyclic compounds, the concentrations of thiophenes (primarily 2-pentylthiophene) and furans (2-pentylfuran and 2-ethylfuran) significantly increased with ultrasonic intensity. These compounds, with their low odor thresholds, made significant contributions to the overall flavor, and their formation was closely related to the MR-lipid interactions promoted by ultrasonication (Hu et al., 2010). Notably, 3-ethyl-2,5-dimethylpyrazine detected in pyrazines was a key component of roasted aroma, and its concentration was positively correlated with ultrasonic intensity, confirming that ultrasonic treatment promoted the formation of pyrazine precursors in the samples, leading to the generation of a large amount of pyrazines during heating (Adan et al., 2025).

The changing pattern of esters further revealed the promoting effect of ultrasonication. Three types of esters, primarily fatty acid esters, were identified in the WP models. Ultrasonic pretreatment induced oxidation and hydrolysis of residual fatty acyl groups. This accelerated the esterification reaction between released free fatty acids and alcohols. Consequently, concentrations of esters such as octyl formate and phenylethyl hexanoate increased. These increases endowed the WPs with rich roasted and fruity aromas typical of animal foods (Szudera-Kończal et al., 2023). Meanwhile, as the most abundant organic acid in the flavor samples, the increase in acetic acid content corresponded with a decreasing pH trend, reflecting the accelerated progression of MR and lipid degradation in the samples promoted by ultrasonic treatment.

### 3.9 Effect of ultrasonic pretreatment on flavor components and sensory properties: A multivariate analysis

To elucidate the key chemical changes responsible for the improved sensory properties, particularly debittering, we performed multivariate analyses. PLS-DA and OPLS-DA models (Fig. 4D-I) were constructed using the composition of volatile compounds and free amino acids as X variables, and the sensory scores (especially bitterness, umami, and roasted aroma) as Y variables. The PLS-DA model showed clear separation between the control (U0–120) and ultrasound-treated groups (U15–120, U30–120, U45–120), with U30–120 and U45–120 forming a distinct cluster. The VIP scores identified the key compounds driving this separation. Notably, bitter free amino acids (e.g., Valine, Leucine, Phenylalanine) were identified as having high VIP scores, confirming their role in bitterness. The model further revealed that their concentrations were negatively correlated with the reduction in bitterness scores, validating the debittering effect of US-MR. In contrast, compounds such as pyrazines (3-ethyl-2,5-dimethylpyrazine), esters, and specific aldehydes (e.g., (E)-2-octenal) had high VIP scores and were positively correlated with the improved sensory attributes of the ultrasound-treated groups, such as roasted aroma, umami, and flavor intensity. This analysis provides a clear chemical basis for the debittering and flavor enhancement observed, linking the consumption of bitter free amino acids and the formation of pleasant aroma compounds to the overall improvement in sensory quality.

### 3.10 Effect of ultrasonic pretreatment on sensory evaluation

**Fig. 5:**
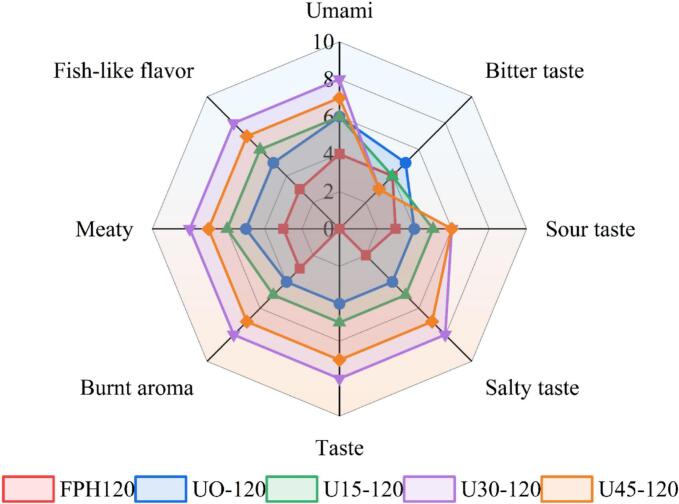
Sensory analysis of peptides: Taste radar chart.

Descriptive sensory analysis was performed to evaluate the sensory attributes of the flavor samples, and the results are presented in Fig. 5. Ultrasonic pretreatment exerted a significant influence on all sensory properties of the flavor model, including roasted aroma, fishy odor, meaty aroma, umami, mouthfeel, sourness, saltiness, flavor intensity, and bitterness. Among the samples, U30–120 and U45–120 exhibited the most intense overall flavor characteristics, which were closely associated with their higher contents of aldehydes, ketones, and alcohols. Concurrently, these two samples displayed a more prominent roasted aroma, primarily attributed to their abundant pyrazine and ester compounds. Notably, the U0–120 sample presented the strongest meaty aroma. This was correlated with its specific content of (E)-1-octen-3-ol, heterocyclic compound with a characteristic meaty flavor profile, which was more abundant in this sample. Compared with the control sample, the alcohol content significantly increased after applying 30% and 45% ultrasonic amplitudes (*p* ≤ 0.05). Most alcohols, such as 1-octen-3-ol, 1-heptanol, and 2-dec-1-ol, were only detected in the samples after ultrasonic treatment. This might be due to the ultrasonic pretreatment promoting the degradation of unsaturated fatty acids. Among them, 1-octen-3-ol had the highest content and was the key component that gave the sample the aroma of mushrooms, citrus fruits, and fats. With the increase in ultrasonic amplitude, the alcohol content in the flavor model significantly increased (p ≤ 0.05), and the alcohol content of U30–120 sample was the highest. In contrast, the sugar- and cysteine-free FPH120 sample (subjected only to thermal treatment) exhibited the highest bitterness intensity. In terms of taste attributes, U30–120 and U45–120 received high scores for umami, saltiness, and mouthfeel richness. This is likely because ultrasonic treatment promoted the cross-linking of peptide fragments with molecular weights ranging from 500 to 1000 Da, thereby enhancing these taste characteristics (Guo et al., 2025). Furthermore, the bitterness intensity of U30–120 and U45–120 was significantly reduced, primarily due to the conversion and consumption of bitter peptides and bitter amino acids during the US-MR (Geng et al., 2019). Meanwhile, sourness became more pronounced with increasing ultrasonic intensity, reflecting an elevation in the organic acid content of the system.Overall, ultrasonic pretreatment effectively suppressed the bitterness of WP products while significantly enhancing their umami, mouthfeel, and sourness attributes, thereby achieving comprehensive optimization of sensory quality.

### 3.11 Effect of ultrasonic pretreatment on antioxidant activity

**Fig. 6:**
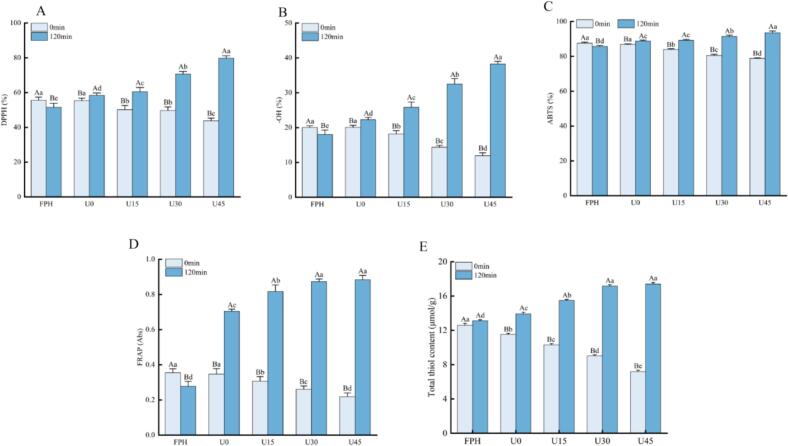
Antioxidant activity of WP. (A) DPPH radical scavenging activity; (B) hydroxyl radical scavenging activity; (C) ABTS radical scavenging activity; (D) reducing power, (E) Total thiol content. For the same heating temperature (120 °C), different lowercase superscript letters above the bars indicate significant differences (*p* < 0.05).

The antioxidant capacities of both unheated and heated flavor model systems were comprehensively assessed using four classic assays, namely DPPH radical scavenging, -OH radical scavenging, ABTS radical scavenging, and reducing power assays (Fig. 6A-D). The results demonstrated that the antioxidant activity of sugar-free FPH samples decreased significantly (*p* < 0.05) after thermal treatment, which could be ascribed to the thermal degradation of intrinsic antioxidant peptides during heating. For the unheated WP samples subjected to ultrasonic pretreatment (U15, U30, and U45), their antioxidant activity showed a gradual decline with the elevation of ultrasound amplitude. This trend is presumably associated with the oxidation of cysteine thiol groups triggered by ultrasonic effects. To verify this viewpoint, we measured the total thiol content (Fig. 6E). The control group had a thiol content of 12.56 μmol/g, while U30 and U45 decreased to 9.02 and 7.19 μmol/g respectively, confirming that ultrasound oxidized the thiol groups. Independent ultrasound (without heating) disrupted the active structural integrity of the peptide chain through mechanical shearing and cavitation effects, resulting in thiol oxidation, a decrease in total thiol content, and a decline in external antioxidant activity. In contrast, ultrasound combined with 120 °C high-temperature treatment could open the compact conformation of the peptide and break the disulfide bonds, exposing a large number of buried thiol groups, significantly increasing the total thiol content; at the same time, high temperature reshaped the peptide spatial structure and optimized the distribution of active groups. The abundant free thiol groups significantly enhanced antioxidant activity through hydrogen supply, chelation of metal ions, and neutralization of free radicals. In sharp contrast, all WP samples exhibited a significant enhancement in antioxidant activity (*p* < 0.05) following heating. This improvement is likely derived from the formation of novel bioactive compounds via the MR, including volatile derivatives, low-molecular-weight heterocyclic compounds, and cross-linked peptides. Notably, ultrasonic pretreatment exerted a pronounced positive effect on the antioxidant activity of heated WP samples (*p* < 0.05), with the extent of enhancement showing a positive correlation with ultrasound intensity. This observation indicates a distinct synergistic effect between ultrasonic treatment and the MR in boosting the antioxidant capacity of the system. The underlying mechanisms are multi-faceted: first, the cavitation effect induced by ultrasound accelerates the entire MR process, thereby facilitating the abundant production of antioxidant compounds such as low-molecular-weight heterocyclic substances and cross-linked peptides; second, ultrasound-mediated conformational changes in peptide chains expose more hidden active groups, further contributing to the elevated antioxidant potential. Additionally, the fluorophore compounds generated during the MR are capable of donating hydrogen atoms and can directly participate in free radical scavenging reactions (Li et al., 2021). These findings are consistent with the results obtained from FTIR spectroscopy, absorbance measurements, and fluorescence analyses, collectively verifying that ultrasonic pretreatment promotes the formation of cross-linked peptides, fluorophores, as well as MR intermediate and advanced products during heating. Thus, ultrasonic treatment enhances the overall antioxidant capacity of the system by expediting the MR process. These results are also in line with previous reports, where MRPs were found to significantly improve the antioxidant activity of oyster protein hydrolysates (He et al., 2021). This strategy of coupling physical field treatment with chemical reactions not only offers a novel perspective for elucidating the antioxidant mechanisms of MR products but also paves new avenues for the development of high-efficiency natural antioxidants for food and biomedical applications.

## 4 Conclusion

This study investigated the application of US-MR for the debittering of WP. A systematic evaluation demonstrated that ultrasonic pretreatment-particularly at 30% amplitude-significantly accelerated the Maillard reaction kinetics of WP during thermal processing, thereby inducing more pronounced structural modifications in the peptide moiety. FTIR spectroscopy confirmed that ultrasonic pretreatment facilitated covalent cross-linking between walnut peptides and glucose. Compared with the untreated control and other treatment groups, the 30%-amplitude US-MR treatment yielded a greater reduction in bitter free amino acids, higher enrichment of key volatile flavor compounds-including aldehydes, ketones, and pyrazines-and enhanced antioxidant activity. Although ultrasonic pretreatment at 45% amplitude also improved several quality attributes, a decline in certain indicators was observed, suggesting suboptimal conditions. Sensory evaluation further corroborated that US-MR at 30% amplitude most effectively enhanced WP's aroma, umami taste, and mouthfeel while concurrently mitigating bitterness. In conclusion, US-MR processing-with 30% ultrasonic amplitude identified as the optimal parameter-represents a promising strategy for producing high-quality, naturally debittered walnut peptides.

## CRediT authorship contribution statement

**Xuehang Wang:** Writing – original draft, Methodology, Conceptualization. **Xinyue Li:** Software, Formal analysis, Data curation. **Mingkai Sun:** Visualization, Validation, Supervision. **Hengwen Liu:** Resources. **Yue Leng:** Methodology. **Bin Jiang:** Formal analysis. **Jingqi Zhao:** Investigation. **Dayong Ren:** Project administration, Conceptualization. **Ji Wang:** Writing – review & editing, Funding acquisition.

## Ethics statement

All sensory panelists were healthy adult volunteers from the university community who provided written informed consent prior to testing, with full disclosure of the study purpose, food sample nature, non-invasive procedures, and right to withdraw without penalty. No personal identifying information was recorded to protect privacy. As the sensory evaluation only involved routine, low-risk assessments of conventional food products without invasive procedures, biological sample collection, or vulnerable populations, formal Institutional Review Board (IRB) ethical approval was not required, and all procedures complied with the Declaration of Helsinki's ethical principles.

## Declaration of competing interest

The authors declare that they have no known competing financial interests or personal relationships that could have appeared to influence the work reported in this paper.

## Data Availability

The data that has been used is confidential.
